# The decomposition process and nutrient release of invasive plant litter regulated by nutrient enrichment and water level change

**DOI:** 10.1371/journal.pone.0250880

**Published:** 2021-05-03

**Authors:** Ruirui Yang, Junyu Dong, Changchao Li, Lifei Wang, Quan Quan, Jian Liu

**Affiliations:** 1 Environment Research Institute, Shandong University, Qingdao, China; 2 State Key Laboratory of Eco-hydraulics in Northwest Arid Region of China, Xi’an University of Technology, Xi’an, China; Sichuan Agricultural University, CHINA

## Abstract

Wetlands are vulnerable to plant invasions and the decomposition of invasive plant litter could make impacts on the ecosystem services of wetlands including nutrient cycle and carbon sequestration. However, few studies have explored the effects of nutrient enrichment and water level change on the decomposition of invasive plant litter. In this study, we conducted a control experiment using the litterbag method to compare the decomposition rates and nutrient release in the litter of an invasive plant *Alternanthera philoxeroides* in three water levels and two nutrient enrichment treatments. This study found that the water level change and nutrient enrichment showed significant effects on the litter decomposition and nutrient dynamic of *A*. *philoxeroides*. The increase of water level significantly reduced the decomposition rate and nutrient release of litter in the nutrient control treatment, whereas no clear relationship was observed in the nutrient enrichment treatment, indicating that the effect of water level change on litter decomposition might be affected by nutrient enrichment. At the late stage of decomposition, the increase of phosphorus (P) concentration and the decrease of the ratio of carbon to P suggested that the decomposition of invasive plant litter was limited by P. Our results suggest that controlling P enrichment in water bodies is essential for the management of invasive plant and carbon sequestration of wetlands. In addition, the new index we proposed could provide a basis for quantifying the impact of invasive plant litter decomposition on carbon cycle in wetlands.

## Introduction

As important components of terrestrial carbon pool, wetlands are closely related to global climate change [1, 2]. Plant litter is the major input of soil organic carbon in wetlands that has a direct impact on the formation and turnover of soil organic matter in wetlands [3–6]. Also, plant litter indirectly impacts the nutrient cycle and carbon sequestration in wetlands through its effects on soil environment, plant, and microbial communities [7, 8]. Wetlands are vulnerable to plant invasions and the native plants in wetlands are easily replaced by the invasive plants [9, 10]. Because of the distinct traits of invasive plants such as high litter quality and high biomass [11–13], the litter decomposition of invasive plants always has critical impacts on the nutrient cycle and soil organic matter in wetlands [14, 15]. Therefore, it is essential to study the litter decomposition process of invasive plants for understanding and evaluating the ecological functions of nutrient cycle and carbon sequestration in wetlands.

The litter decomposition of invasive plants in wetlands could be regulated by several external factors like physical and chemical conditions in water [16, 17] and intrinsic factors like initial concentrations of nutrients [18, 19]. In wetland ecosystems, hydrology is an important factor affecting the litter decomposition [20]. As one of the important characteristics of hydrology, water level could be increased by global warming and seasonal rainfall [21, 22]. The increase of water level has been reported limiting the litter decomposition in the wetland ecosystems by altering the aeration condition and restricting the metabolism of microbes [16, 23]. Wallis and Raulings [24] also reported that the fast leaching of dissolved organic matter could result in rapid litter decomposition in the shallow water. However, few studies have considered the nutrient conditions in the decomposition environment when exploring the impacts of increasing water level on litter decomposition [25].

Increasing anthropogenic activities including agriculture, industry, and urbanization accompanied by the increasing utilization of water resources could alter the chemical factors of hydrology environment and result in nutrient enrichment in wetland ecosystems [26–28]. Sun et al. [25] and Scott et al. [26] found that the nutrients status in the decomposition environment could influence the decomposition rates and nutrient dynamic of plant litter. The addition of nitrogen (N) could decrease the ratio of carbon to nitrogen (C/N) and phosphorus (P) concentration, thus accelerating the decomposition of litter [29–31]. Similarly, several studies have reported that the P enrichment in water could increase the P concentration and reduce the ratios of carbon to phosphorus (C/P) and nitrogen to phosphorus (N/P) in litter [26, 32]. The nutrient enrichment in decomposition environment could impact the metabolism of microbes and result in the alternation of litter decomposition and litter stoichiometry [33]. To maintain the stoichiometric balance required for growth, microbes tend to uptake inorganic nutrients from environments [34–36]. Suberkropp et al. [37] found that the increases of N and P in the environment could enhance microbial activities during litter decomposition. Additionally, the addition of carbon (C) and N could affect plant litter decomposition by altering microbial respiration [38], and the availability of C limited microbial biomass [39]. The metabolism of microbes related to litter decomposition could be affected by water level change [16, 24]. However, few investigators have researched the interaction of water level change and nutrient enrichment on litter decomposition including nutrient release and stoichiometry. The study on the effects of water level change and nutrient enrichment on litter decomposition is important for predicting the impacts of nutrient enrichment on carbon sequestration in wetlands under global climate change.

*Alternanthera philoxeroides*, originated in South America, has invaded many countries and regions as a malignant invasive plant [40, 41]. *A*. *philoxeroides* was first introduced in China in 1930s and invaded in most regions of south China [42, 43]. Previous studies have reported that *A*. *philoxeroides* produced higher quality litter with faster decomposition rate than the native species [44, 45], which could make crucial impacts on the nutrient cycle and carbon sequestration in wetland ecosystems [46]. Therefore, we took *A*. *philoxeroides* as an example to conduct the control experiment of nutrient enrichment and water level change. In this study, we first hypothesized that the decomposition rate and nutrient release of *A*. *philoxeroides* litter showed significant difference in different water levels and nutrient enrichment treatments. Secondly, the effects of water level change on the decomposition process of *A*. *philoxeroides* litter were affected by nutrient enrichment. To test these hypotheses, we studied the variation of litter mass and nutrient dynamic during the decomposition process. The decomposition rate (K) of litter, derived from the negative exponential model proposed by Olson [47], is used to show the speed of decomposition after a period of time [31, 48]. Previous study has reported that decomposition rate could not indicate the integrity of decomposition and the effects of environmental factors on the whole decomposition process [49]. Due to the limitation of decomposition rate, we also proposed a new index of real-time decomposition rate to reflect the effects of water level change and nutrient enrichment on the decomposition process of *A*. *philoxeroides* litter.

## Materials and methods

### Litter collection and experimental design

#### Litter collection and pretreatment

Because the invasion of *A*. *philoxeroides* into wetlands always forms a dense single species, we conducted a study for the decomposition of single-species litter. In November 2018, the litter of *A*. *philoxeroides*, consisting of leaves and stems, were collected from Xinxue River Constructed Wetland in Nansi Lake (34° 27′−35° 20′E, 116° 34′−117° 21′N), Shandong Province. The collected litter was dried in an oven at 65°C to a constant weight followed many related studies [19, 50]. The dried plants were cut into 10 cm long and weighed about 20 g as one sample.

#### Ethics statements

The Management Committee of the Xinxue River Constructed Wetland approved the sample collection of *A*. *philoxeroides* litter in this study. There was no protected species were sampled in this study.

#### Experimental design

As one of the most common methods for the determination of litter nutrient dynamics and mass remaining during the decomposition, the litterbag method was used for the control experiment [51]. Approximately 20 g of plant litter was packed in a 20 cm×25 cm nylon bag (0.3 mm mesh), weighed, and labeled. All the samples were placed into plastic buckets and fixed at the interface of water and air (diameter 30 cm × height 30 cm). *A*. *philoxeroides* litter decomposed in three different water levels and two nutrient enrichment treatments. According to previous studies and the field investigation on water level, the three water levels in the experiment were set as 5 cm, 15 cm, and 25 cm, representing the low, middle, and high water levels, respectively [22, 52]. The control treatment of nutrient enrichment was the tap water, and the nutrient enrichment treatment was the synthetic wastewater (S1 Text, S1 Table) which simulated the wastewater directly discharged into the river after being treated by sewage treatment plants [53]. According to water levels, the nutrient control treatment and the nutrient enrichment treatment were set using the plastic buckets. To inoculate microbes, we added the fresh marsh water from Xinxue River Constructed Wetland into each plastic buckets in proportion, and then placed all the plastic buckets in a dark room with the temperature of 20 ~ 25°C [54]. Since the half-life of aquatic plants is generally 17 ~ 58 days [55], the litterbags of *A*. *philoxeroides* litter were collected 7, 14, 21, 28, 42, 56, and 70 days after the beginning of litter decomposition, 5 untreated samples were also collected to get the initial concentrations of nutrients. We had 3 water levels × (nutrient control treatment + nutrient enrichment treatment) × 7 time samplings × 5 replicates + 5 untreated samples = 215 samples.

### Laboratory analysis

The litter in retrieved litterbags was washed with pure water and dried to a constant weight in the oven at 65°C [19]. The remaining litter was weighed, ground, sieved by 0.21 mm sieve, and then stored in sealed bags prior to chemical analyses. The C and N concentrations of the remaining litter were determined by an elemental analyzer (Vario EL III, Elementar Analysensysteme GmbH, Germany). The concentration of P was measured by the method described by Lu [56] using a spectrophotometer (UV-2450, Shimadzu Scientific Instruments, Japan).

### Data analysis

#### Mass remaining and nutrient release

The mass remaining of litter after a period time of decomposition was calculated following the equation [57]:

$$Mass\;Remaining\left(\%\right)=\frac{M_{t}}{M_{0}}\times 100$$ (1)

where M_t_ represents the dry weight of litter remaining after t time of decomposition, M_0_ means the initial weight of litter.

The nutrient release of litter after a period time of decomposition was calculated following the equation [58]:

$$Nutrient\;Release\left(\%\right)=\frac{{M_{0}C_{0}-M}_{t}C_{t}}{M_{0}C_{0}}\times 100$$ (2)

where M_t_ represents the dry weight of litter remaining after t time of decomposition, M_0_ means the initial weight of litter, C_t_ represents the nutrient concentration in the remaining litter at time t, C_0_ is the initial concentration of the litter nutrient.

#### The decomposition rate and the real-time decomposition rate

The decomposition rate (K) of litter was fitted to the negative exponential decomposition model [47]:

$$\frac{M_{t}}{M_{0}}=e^{-Kt}$$ (3)

where M_t_ represents the dry weight of litter remaining after t time decomposition, M_0_ means the initial weight of litter, t is the time (day), K represents the decomposition rate after t time of decomposition.

The new index of real-time decomposition rate (K_i_) we proposed was also fitted to the negative exponential decomposition model:

$$\frac{M_{i}}{M_{i-1}}=e^{-K_{i}}$$ (4)

where M_i_ represents the dry weight of litter remaining after i time of decomposition, M_i-1_ means the dry weight of litter remaining after i-1 time of decomposition, i and i-1 are the time points (day), K_i_ is regarded as the decomposition rate at time i. The derivation of this formula is in the S1 Text.

#### Statistical analysis

All statistical analyses were carried out using SPSS 22.0 and the data were shown in figures drawn by Origin 2017. The effect of water level change, nutrient enrichment, and their interactions on litter mass remaining, nutrient concentration and release, elements (C, N, P) stoichiometry, decomposition rate (K), and real-time decomposition rate (K_i_) were examined by one-way and two-way analysis of variance (ANOVA) with LSD test. Spearman correlations were conducted for the relationships between real-time decomposition rate (K_i_) and the nutrient concentration and elements stoichiometry. Using the nutrient concentration and elements stoichiometry of decomposing litter as independent variables, stepwise multiple regression analyses were carried out to evaluate the best predictors of the real-time decomposition rate (K_i_) and C, N, P released. We calculated the ratio of C release to N release to show the degree of coupling relation between C and N release, and tested the significant difference of the ratio among the experimental treatments using one-way ANOVA. A stepwise multiple regression analysis was performed to assess the factors affecting the coupling relationship between C and N release during the litter decomposition.

## Results

### Mass remaining during litter decomposition

**Fig 1:**
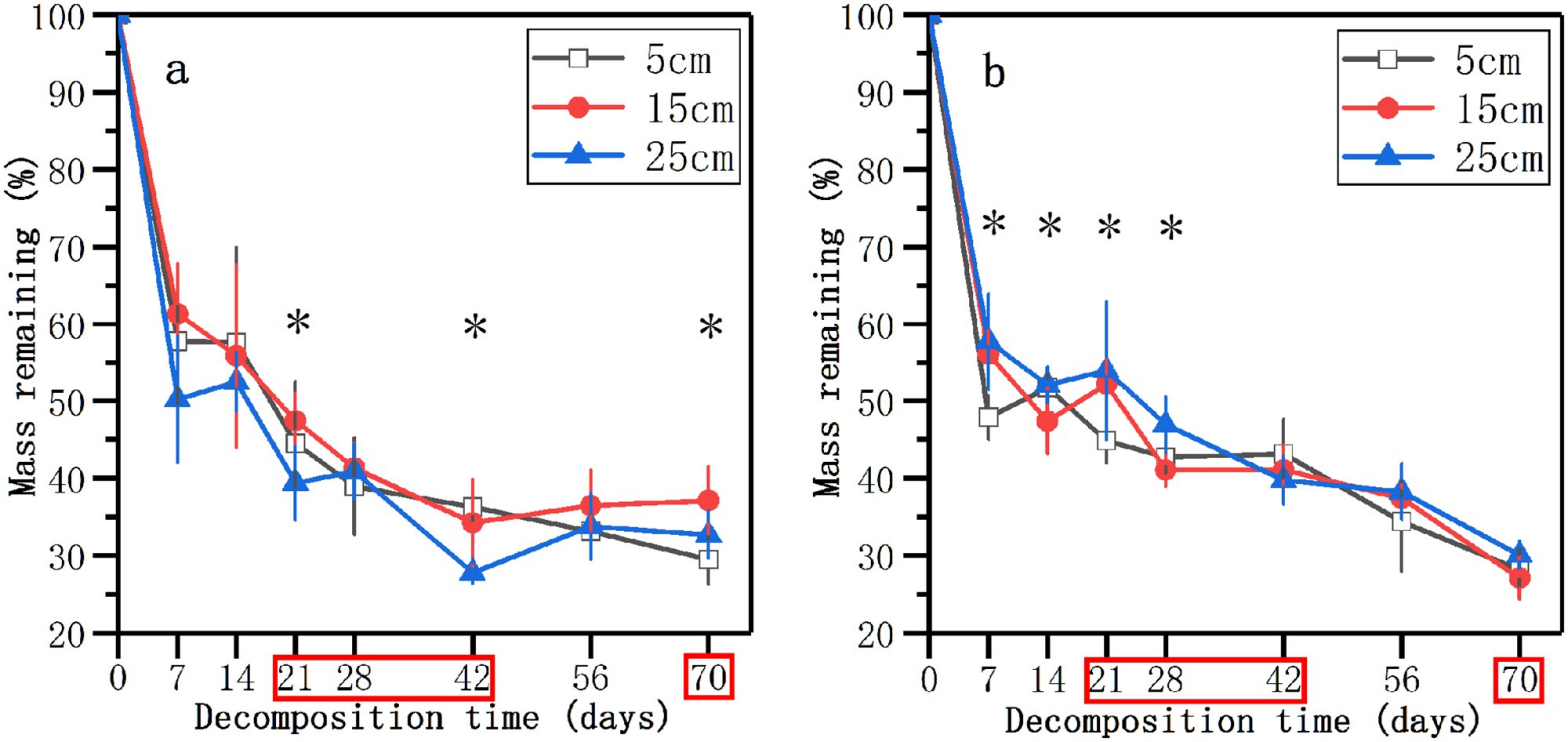
Mass remaining weights (%) of decomposing litter in three water levels in the nutrient control treatment (a) and nutrient enrichment treatment (b) during the experimental period. Data represent means ± SD (n = 5). * indicates significant differences at *p* = 0.05 level in different water levels, the time points in the red box indicate significant differences at *p* = 0.05 level in different nutrient enrichment treatments, LSD test.

Mass remaining decreased sharply within the initial 7-day incubation, and then decreased slowly (Fig 1). After 7 days, there was an evident interaction between nutrient enrichment and water level on the mass remaining. The remaining weight of litter in 25 cm water level in the nutrient control treatment was lower than those in other water levels (Fig 1a), while the remaining weight was significantly higher in 25 cm water level in the nutrient enrichment treatment (*p* < 0.05, Fig 1a). In the nutrient control treatment, the mass remaining in different water levels showed significant differences at 21, 42, and 70 days (*p* < 0.05, Fig 1a). However, the significant differences in the mass remaining between different water levels existed at 7, 14, 21, and 28 days in the nutrient enrichment treatment (*p* < 0.05, Fig 1b). At 21, 28, 42, and 70 days, there were significant differences in the remaining weight of litter between the nutrient control treatment and nutrient enrichment treatment (*p* < 0.05, Fig 1). After 70 days of decomposition, the mass remaining in all three water levels in the nutrient control treatment was higher than those in the nutrient enrichment treatment. At 70 days, the remaining weight of litter in 15 cm level in the nutrient control treatment and the nutrient enrichment treatment showed significant differences at 37.1% and 27.1% (*p* < 0.05, Fig 1).

### Carbon, nitrogen, and phosphorus release and litter stoichiometry

**Fig 2:**
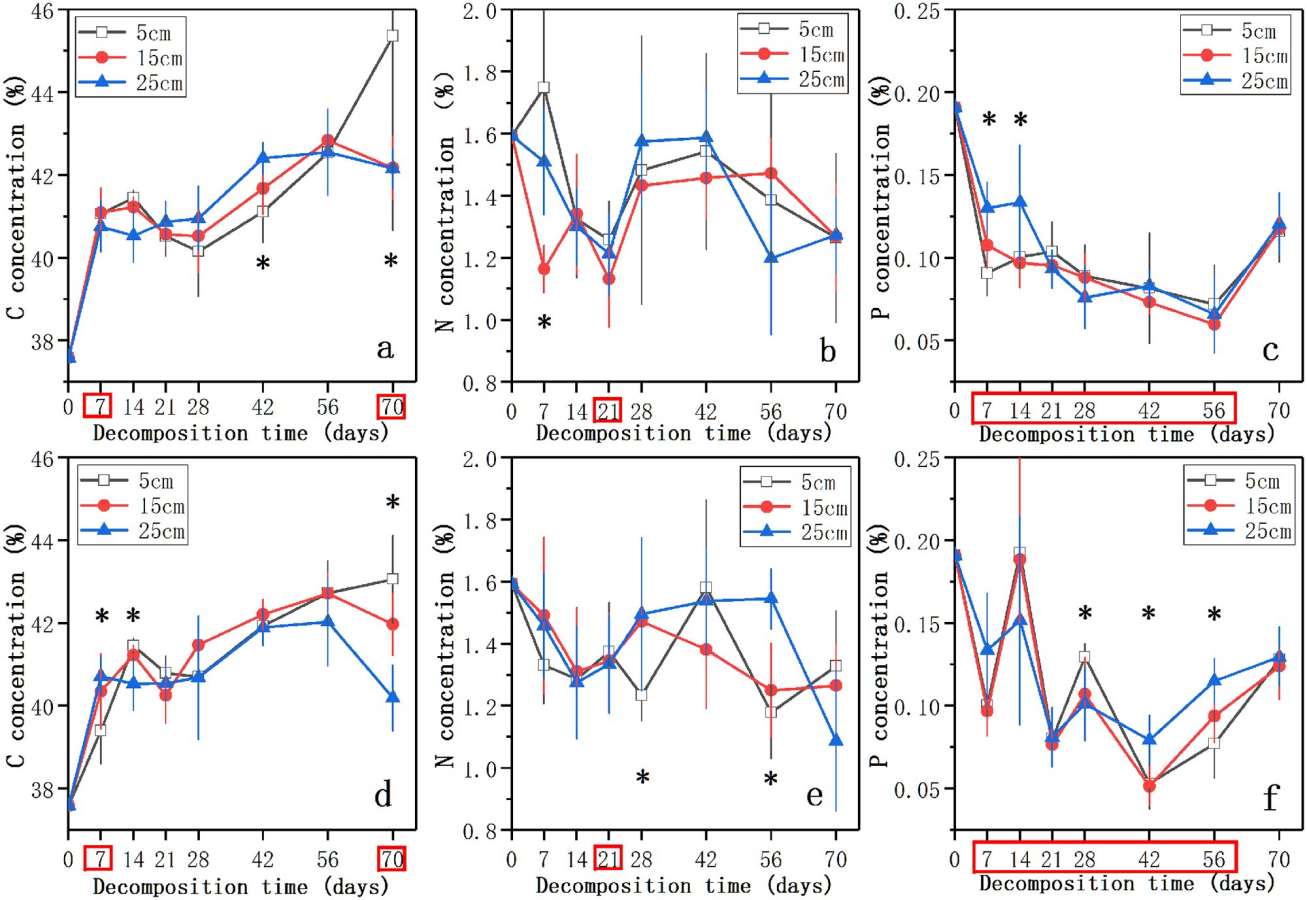
The dynamics of decomposing litter nutrient concentrations (%) of C, N, and P in the three water levels in the nutrient control treatment (a, b, c) and the nutrient enrichment treatment (d, e, f) during the experimental period. Data represent means ± SD (n = 5). * indicates significant differences at *p* = 0.05 level in different water levels, the time points in the red box indicate significant differences at *p* = 0.05 level in different nutrient enrichment treatments, LSD test.

Fig 2 showed the dynamics of C, N, and P concentrations in the three water levels in the nutrient control treatment and the nutrient enrichment treatment during the process of litter decomposition. The initial concentrations of C, N, and P were 37.59%, 1.59%, and 0.19%, respectively. The C concentration of decomposing litter in each water level increased with the decomposition time until 56 days (Fig 2). After that, the C concentration in 15 cm water level and 25 cm water level decreased, while that in 5 cm water level continued to increase. At 70 days, the C concentration in 5 cm level in the nutrient control treatment and the nutrient enrichment treatment was significantly higher than those in 15 cm and 25 cm levels (*p* < 0.05, Fig 2a and 2d). In each water level, the C concentration of litter was higher in the nutrient control treatment than in the nutrient enrichment treatment (Fig 2a and 2d). Except 5 cm water level in the nutrient control treatment, the N concentration of litter in other treatments first decreased, and then increased until 42 ~ 56 days followed by a decline thereafter (Fig 2b and 2e). After 70 days, the N concentration in the nutrient control treatment decreased to 1.26 ~ 1.27% and showed no significant difference (*p* > 0.05, Fig 2b). In the nutrient enrichment treatment, the N concentration at 70 days decreased in the following order: 5 cm > 15 cm > 25 cm (Fig 2e). Nutrient enrichment showed significant effect on the N concentration only at 21 days (*p* < 0.05, Fig 2b and 2e). In addition, the concentration of P in decomposing litter showed an overall trend of decline followed by rise, and the P concentration in the nutrient enrichment treatment showed more violent changes than that in the nutrient control treatment (Fig 2c and 2f). In the nutrient control treatment, the significant differences in P concentration between different water levels were existed at 7 and 14 days (*p* < 0.05, Fig 2c). However, the time points when water level change had a significant effect on the P concentration of decomposing litter in the nutrient enrichment treatment were at 28, 42, and 56 days (*p* < 0.05, Fig 2f). The P concentration of litter showed significant difference between the control treatment and nutrient enrichment treatment at 7, 14, 21, 28, 42, and 56 days (*p* < 0.05, Fig 2c and 2f). After 70 days, the P concentration of litter decreased to 0.11 ~ 0.13% and showed difference in the three water levels, while the P concentration in each water level in the nutrient enrichment treatment was higher than that in the nutrient control treatment (Fig 2c and 2f). The immobilization period of C, N, P concentrations was not detected during the process of litter decomposition.

**Fig 3:**
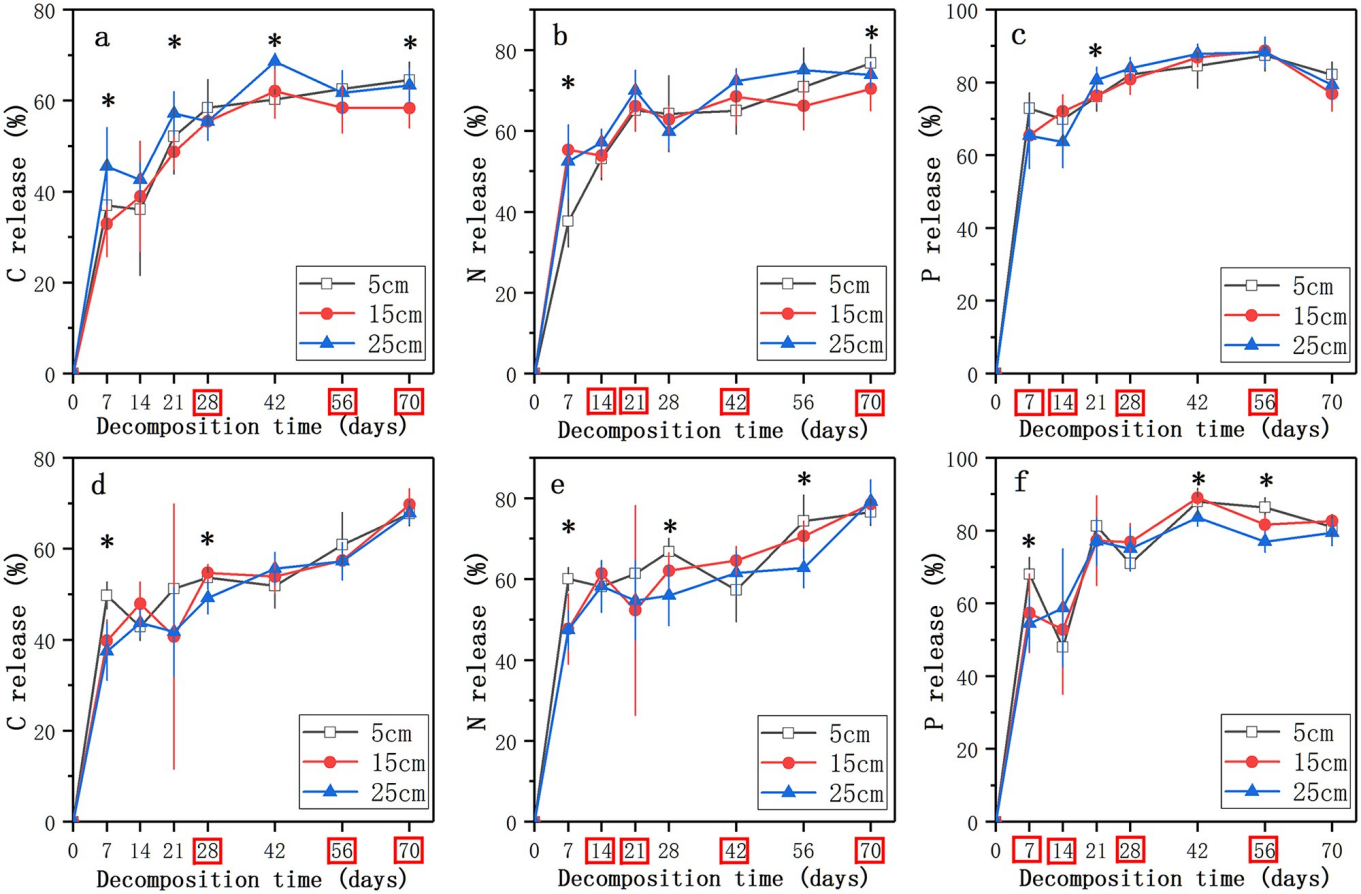
The nutrients release (%) of decomposing litter in the three water levels in the nutrient control treatment (a, b, c) and the nutrient enrichment treatment (d, e, f) during the experimental period. Data represent means ± SD (n = 5). * indicates significant differences at *p* = 0.05 level in different water levels, the time points in the red box indicates significant differences at *p* = 0.05 level in different nutrient enrichment treatments, LSD test.

The nutrient release of decomposing litter including C and N increased in the same trend over the study period, while the P release showed a trend of increasing first and then decreasing (Fig 3). The significant effect of water level on C release in the nutrient enrichment treatment was at the early stage of decomposition process (14 and 28 days), while it existed in the whole process in the nutrient control treatment (*p* < 0.05, Fig 3a and 3d). At the end of litter decomposition, percentages of C release in 5 cm, 15 cm, and 25 cm water level in the nutrient control treatment were 64.5%, 58.4%, and 63.3%, and percentages of C release in the three water levels in the nutrient enrichment treatment were 67.8%, 69.7%, and 67.8%, respectively (Fig 3a and 3d). There were significant differences in percentages of C release between 5 cm and 15 cm in the nutrient control treatment (*p* < 0.05, Fig 3a), while there was no significant difference in different water levels in the nutrient enrichment treatment. In 15 cm and 25 cm, C release was significantly lower in the nutrient control treatment than in the nutrient enrichment treatment (*p* < 0.05, Fig 3a and 3d). As for the N release, the percentage at 70 days in 5 cm water levels in the nutrient control treatment was significantly higher than that in 15 cm water level (*p* < 0.05, Fig 3b), while there was no significant difference in the three water levels in the nutrient enrichment treatment (Fig 3e). Similar to C, the percentages of N release in the three levels (76.7% in 5 cm, 70.5% in 15 cm, and 73.9% in 25 cm; Fig 3b) in the nutrient control treatment were lower than those in the nutrient enrichment treatment (76.6% in 5 cm, 78.6% in 15 cm, and 79.3% in 25 cm; *p* < 0.05, Fig 3e), but only in 15 cm level it showed a significant difference in the two types of water (*p* < 0.05, Fig 3b and 3e). After 70 days of decomposition, shifts in P release in the nutrient control treatment and the nutrient enrichment treatment were consistent with that in N release (Fig 3c and 3f), but there was no significant difference in the three water levels.

**Fig 4:**
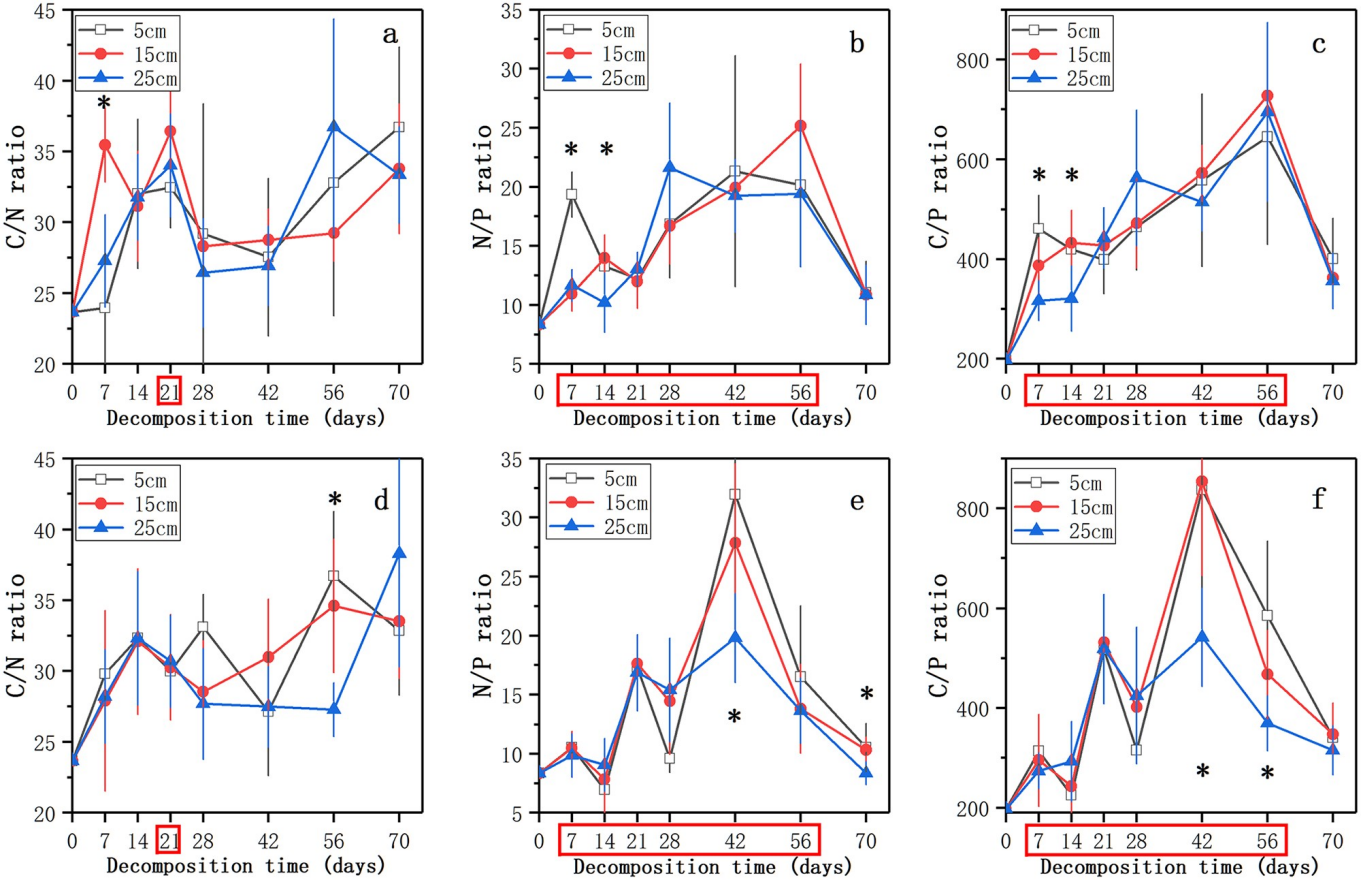
Changes in the C/N (a, d), N/P (b, f), and C/P (c, e) ratios of decomposing litter in the nutrient control treatment (a, b, c) and the nutrient enrichment treatment (d, e, f) during the experimental period. Data represent means ± SD (n = 5). * indicates significant differences at *p* = 0.05 level in different water levels, the time points in the red box indicate significant differences at *p* = 0.05 level in different nutrient enrichment treatments, LSD test.

The trend of the C/N ratio was first increased, and then decreased until 28 ~ 42 days followed by a decline thereafter. After 70 days, the C/N ratio of litter increased from 23.60 to 32.86 ~ 38.29 (Fig 4a and 4d). The N/P ratio in the nutrient control treatment increased until 56 days and decreased to 10.85 ~ 11.05 thereafter (Fig 4b). And in the nutrient enrichment treatment, the N/P ratio from intial to 42 days and then decreased, it was significantly higher in 5 cm level at 70 days than in 25 cm (*p* < 0.05, Fig 4e). The C/P ratio of litter showed the same trend with N/P ratio, and the time points when nutrient enrichment had a significant effect on the N/P and C/P ratios of decomposing litter were the same with the P concentration (*p* < 0.05, Fig 4). After 70 days of decomposition, the litter in the three water levels in the nutrient enrichment treatment had a lower N/P and C/P ratios than that in the nutrient control treatment (Fig 4).

Results of stepwise-regression analyses of litter nutrient release with the nutrient concentration and stoichiometric ratios of litter were showed in S2 Table. During the litter decomposition, C, N, and P release of litter were all controlled by the C concentration, P concentration, C/N ratio, and C/P ratio of decomposing litter (*p* < 0.001, S2 Table).

### Litter decomposition rate and real-time decomposition rate

The decomposition rate (K, day^-1^) of litter in each treatment was highest at 7 days than other time points. The results of two-way ANOVA showed a significant interaction between water level and nutrient enrichment on the decomposition rate after 7 days (Table 1). In the nutrient control treatment, the decomposition rate of litter was higher in 25 cm water level than in other two water levels, while the decomposition rate in the nutrient enrichment treatment had significantly higher value in 5 cm water level than in 15 cm and 25 cm (*p* < 0.05, Table 1). From 7 days to 70 days, the decomposition rates of litter in the three levels in the nutrient control treatment and the nutrient enrichment treatment gradually declined. At 70 days, litter decomposition rate in the nutrient control treatment decreased in the following order: 5 cm > 25 cm > 15 cm (*p* < 0.05, Table 1), while the order of decomposition rate was different in the nutrient enrichment treatment. After 70 days of decomposition, nutrient enrichment had significant effects on the decomposition rate of plant litter (*p* < 0.01, Table 1). In each water level, the decomposition rate in the nutrient enrichment treatment was higher than that in the control treatment.

**Table 1:** Decomposition rate (K, day^-1^) of plant litter after 7 and 70 days of decomposition and the ANOVA results of K response to water level change, nutrient enrichment and their interactions.

**Nutrient enrichments**	**Water levels (cm)**	**Decomposition rate (K)**	
		7 days	70 days
**Nutrient Control treatment**	5	0.081±0.024	0.018±0.002^Aa^
	15	0.071±0.016	0.014±0.002^Ab^
	25	0.100±0.024	0.016±0.001^Aab^
**Nutrient enrichment treatment**	5	0.105±0.009^a^	0.019±0.001^Aa^
	15	0.083±0.009^b^	0.019±0.002^Ba^
	25	0.079±0.015^b^	0.017±0.001^Aa^
		**F**	
		7 days	70 days
**Water level**		2.453	3.547*
**Nutrient enrichment**		0.706	15.36**
**Water level** * **Nutrient enrichment**		4.674*	3.12

Different uppercase letters indicate significant differences at *p* = 0.05 level in different nutrient enrichment treatments, different lowercase letters indicate significant differences at *p* = 0.05 level in three water levels.

* *p* < 0.05,

** *p* < 0.01, LSD test.

**Fig 5:**
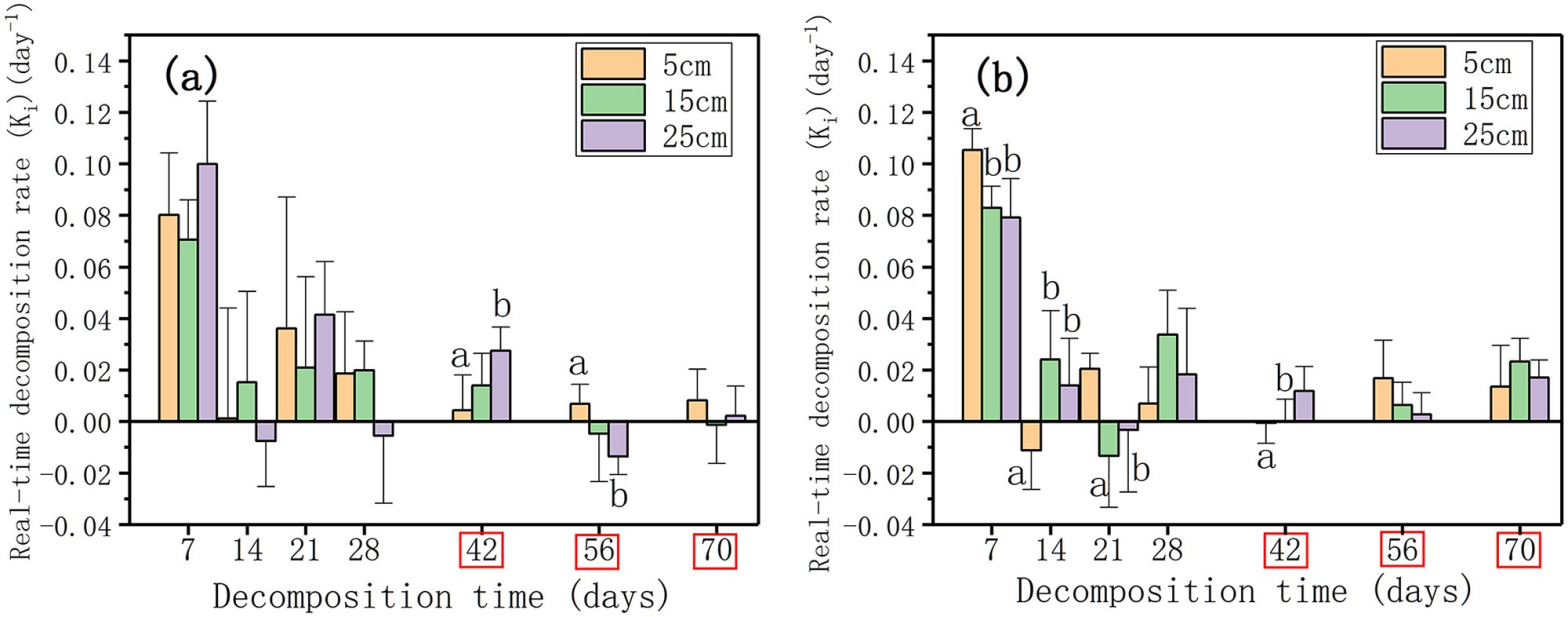
The real-time decomposition rate (K_i_, day^-1^) of litter in three water levels in the nutrient control treatment (a) and the nutrient enrichment treatment (b). Different lowercase letters indicate significant differences at *p* = 0.05 level in different water levels, the time points in the red box indicate significant differences at *p* = 0.05 level in different nutrient enrichment treatments, LSD test.

The real-time decomposition rate (K_i_, day^-1^) of litter showed a downward trend during the decomposition process. The time points when water level change had a significant effect on the real-time decomposition rate of plant litter in the nutrient enrichment treatment were at the early stage (7, 14, 21, and 42 days), while in the nutrient control treatment the time points changed to 42 and 56 days (*p* < 0.05, Fig 5a and 5b). The real-time decomposition rate showed significant difference between the nutrient control treatment and nutrient enrichment treatment at 42, 56, 70 days (*p* < 0.05, Fig 5). After 70 days of decomposition, the real-time decomposition rate in each water level in the nutrient control treatment was less than 0.01 day^-1^ (Fig 5a). The real-time decomposition rates at 70 days had higher values in the nutrient enrichment treatment than in the nutrient control treatment at 0.014 day^-1^ in 5 cm, 0.023 day^-1^ in 15 cm, and 0.017 day^-1^ in 25 cm (*p* < 0.05, Fig 5b).

**Fig 6:**
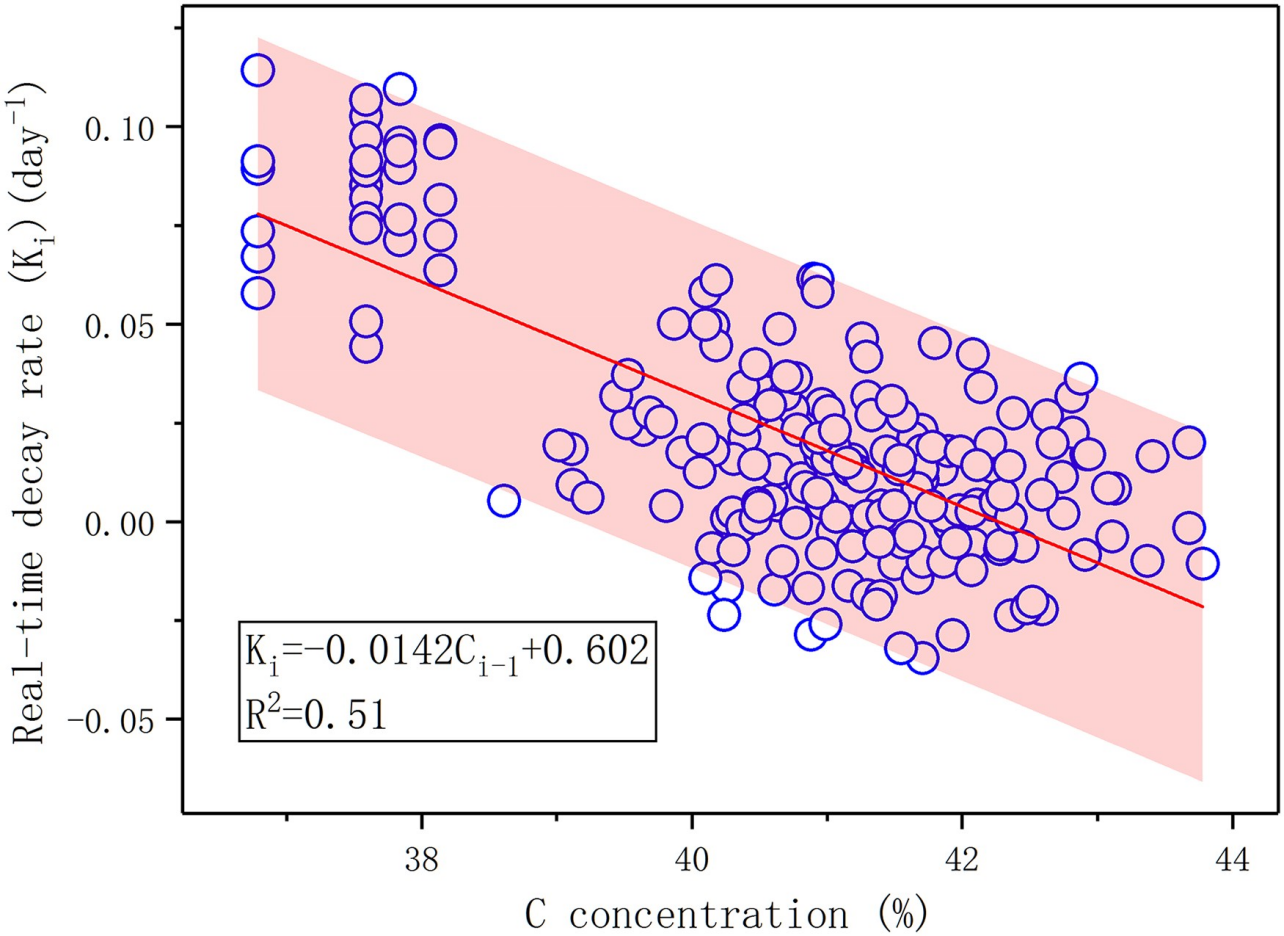
Relationships between real-time decomposition rates at time i (K_i_) and C concentration of the litter at time i-1 in this experiment.

During the decomposition process of litter, the nutrient concentration and stoichiometry of decomposing litter regulated the real-time decomposition rate. Across the control experiment, the real-time decomposition rate at time i had significantly negative correlations with the C concentration, C/N, N/P, and C/P ratios of decomposing litter at time i-1, and a significantly positive correlation with the P concentration of litter at time i-1 (*p* < 0.05, S3 Table). The multiple stepwise-regression analysis revealed that the C concentration at time i-1 best controlled the real-time decomposition rate at time i during the litter decomposition (*p* < 0.001, Fig 6, n = 210). The C concentration explained 51% of the variation in real-time decomposition rate (R^2^ = 0.51, Fig 6).

### The coupling relationship of carbon and nitrogen release during litter decomposition

**Fig 7:**
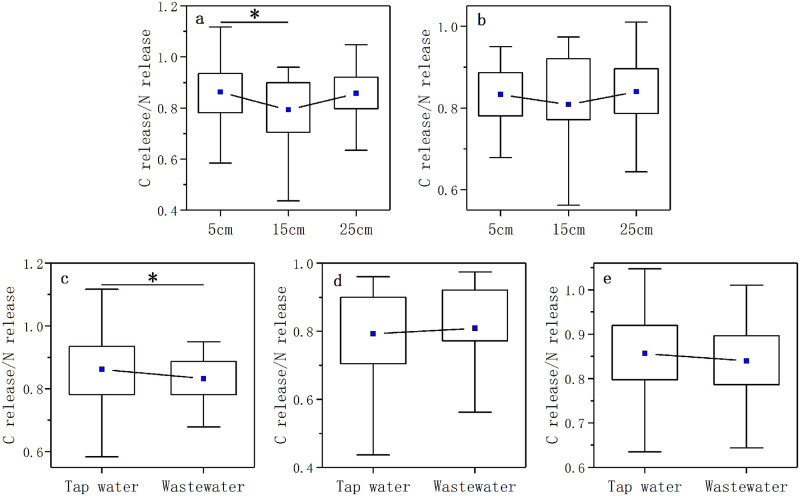
The box diagrams of C release/N release ratio in three water levels in the nutrient control treatment (a) and nutrient enrichment treatment (b). And the box diagrams of C release/N release in two nutrient enrichment treatments in 5 cm (c), 15 cm (d) and 25 cm (e) water levels.

In all the treatments, the C release of decomposing litter showed a linear positive correlation with the N release (S1 Fig). The slope of the linear regression was around 0.85 (0.83–0.86, S1 Fig). The coupling relationship of C release and N release of litter during the process of litter decomposition was shown by the ratio of C release to N release. There was a significant difference in the C release/N release ratio of litter between the 5 cm and 15 cm water levels in the nutrient control treatment (*p* < 0.05, Fig 7a). And the significant difference in the C release/N release ratio of litter also existed in 5cm water level in the two nutrient enrichment treatments (*p* < 0.05, Fig 7c).

The result of stepwise-regression analyses showed N and P concentrations were important variables explained the variation of the coupling relationship between C release and N release (shown by the ratio of C release to N release) (Fig 7). N and P concentrations explained 26.6% of the variation of the ratio of C release to N release (*p* < 0.001, R^2^ = 0.266, Table 2).

**Table 2:** Results of stepwise-regression analyses of C release/N release ratio with the nutrient concentration and stoichiometric ratios of decomposing litter during the litter decomposition process.

Model	Variables	R^2^	*p*	Regressions
**1**	NC	0.250	< 0.001	y = 0.3NC+0.430
**2**	NC, PC	0.266	< 0.001	y = 0.289NC+0.444PC+0.394

NC: N concentration, PC: P concentration.

## Discussion

### Effects of water level change and nutrient enrichment on litter decomposition

Our results demonstrated that water level change had different effects on the invasive plant litter decomposition in the two nutrient enrichment treatments (Fig 1, Table 1). In the nutrient control treatment, the litter in 5 cm water level had significantly lower mass remaining and higher decomposition rate than those in 15 cm water level at the end of decomposition. The rapid decay of litter in the shallow water is consistent with previous studies [25, 59]. The faster rate of leaching nutrients in 5 cm water level which indicated by the lower C concentration (Fig 2) might provide more nutrients for microbial growth and activities and thus accelerated the litter decomposition [24]. In addition, the high water level could result in the low dissolved oxygen or even anaerobic conditions [16, 20]. The decrease of microbe activities under anaerobic conditions might be another explanation for the slow decomposition of litter in the high water level [60]. However, water level change had no significant effect on the litter decomposition in the nutrient enrichment treatment at the end of our experiment, which was different from the nutrient control treatment and previous studies [24, 25]. By comparing the mass remaining and the real-time litter decomposition rate of litter in the different water levels, we found that the effect of water level change on litter decomposition in the nutrient control treatment was mainly at the late stage, while it only existed at the early stage of decomposition in the nutrient enrichment treatment. At the late stage in the nutrient enrichment treatment, the limitation of water level on microbes could be removed by the effect of nutrient enrichment [59, 61]. The result suggested that the effects of water level change on litter decomposition might be affected by the nutrient enrichment in the decomposition environment.

In each water level in this study, the litter in the nutrient enrichment treatment decomposed faster than that in the nutrient control treatment (Table 1), which was in accordance with previous studies [19, 26]. By comparing the real-time decomposition rate of litter in the nutrient enrichment treatment and the nutrient control treatment at 70 days, we found that nutrient enrichment could not only accelerate the decomposition rate of litter, but might even prolong the decomposition process and reduce nutrient residues. The alternation in the litter stoichiometry caused by nutrient enrichment might be reasonable for the acceleration of litter decomposition [25, 26]. The additional N could inhibit the decomposition of lignin and combine with the decomposition products of lignin to form recalcitrant compounds to change the C/N ratio of litter and finally affect litter decomposition [62–64]. The reduced C/P ratio of litter in the nutrient enrichment treatment confirmed the research of Scott et al. which suggested that P enrichment could result in the decrease of C/P ratio [26]. Additionally, the nutrient enrichment in the decomposition environment could cause the response of heterotrophic activities of microbes involved in the litter decomposition, and then further affect the litter decomposition [29, 37, 38]. The higher C concentration of litter in the nutrient control treatment confirmed that microbes in low-nutrient environments could reduce C utilization [36]. Our results demonstrated that nutrient enrichment could accelerate litter decomposition and prolong the decomposition process under the background of global climate change.

The new index of real-time decomposition rate (K_i_) we proposed could show the completeness of litter decomposition and reflect the effects of different environmental factors on the whole decomposition process, and it could be a good supplement to the limitation of decomposition rate [65, 66]. As an important and limiting element for the microbial metabolism in the litter decomposition [36, 67, 68], N concentration has been reported to be significantly positively correlated with litter decomposition rate [69–71]. However, the results of Spearman correlation analysis showed that the limiting factor for the real-time decomposition rate in our study was not N concentration but P concentration (*p* < 0.05, S3 Table). Consistent with the decomposition rate, the real-time decomposition rate also had significantly negative correlation with C concentration and C/N ratio [19, 52, 72]. C/N ratio could reflect the ratio of carbohydrates to proteins which is an essential property of litter [73]. Litter with low C/N ratio is more likely to be ground and decomposed due to lack of structural integrity [74]. Previous studies have reported that C/N ratio was a predictor in litter decomposition rate [19, 71]. Interestingly, the result of multiple regression analysis indicated that C concentration was the dominant predictor of real-time decomposition rate during the litter decomposition process. Compared with the decomposition rate, the real-time decomposition rate can better reflect the influence of different environmental factors on the entire litter decomposition process. Quantifying the real-time decomposition rate can help to understand the plant litter decomposition process in wetland ecosystems in biological and chemical aspects and find the variables for assessing the carbon sequestration function of wetland ecosystems.

### Effects of water level change and nutrient enrichment on nutrient release

Consistent with mass remaining and real-time decomposition rate, the effect of water level on the release of C and N in the nutrient control treatment was at the late stage, while the effect exists in the early stage in the nutrient enrichment treatment. In our research, the results of multiple regression analyses showed that the C and P concentrations, and the C/N and C/P ratios of decomposing litter were the controlling factors of nutrient release (S2 Table). Our results confirmed previous studies [36, 75, 76] that reported the nutrient release of decomposing litter was mainly controlled by litter stoichiometry. By comparing the controlling factors of nutrient release in the nutrient control treatment, we found that the alternation of C concentration might be an explanation for the different nutrient release in the three water levels. The additional P in the nutrient enrichment treatment increased the P concentration and reduced the C/P ratio of litter, which might be reasonable for the different nutrient release in the two nutrient enrichment treatments [26, 32]. Moore et al. [67] and Berg et al. [77] have reported that the net N release of litter was limited by the value of C/N ratio. Throughout the decomposition process of our experiment, the C/N ratio of litter was always less than between 31 and 48 reported by Parton et al. [78], which might be an explanation of the increasing trend of N release. Unlike the trend of N release, the P release trend was increase followed by decrease. The reason for the change in the P release trend might be the alternation of the C/P ratio during the decomposition process [36, 76]. According to the research of Elser et al. [79], the C/P ratio of decomposing litter at the early stage was less than 375, and there was P net release. At the late stage of decomposition, C/P ratio increased more than 400 and P would be fixed in the litter [80]. In addition, the C/P ratio greater than 400 in the late stage proved that the decomposition of invasive plant litter was limited by P in all the treatments [76, 79]. Therefore, controlling the concentration of P in water bodies is of great significance to the management of invasive plant and carbon sequestration of wetlands.

### The coupling relationship between C and N release

In our study, the C release of decomposing litter in all the treatments showed a linear positive correlation with the N release (S1 Fig), while the C release/N release ratio was lower than 1:1 (Fig 7). At the early stage of litter decomposition, microbes could use unstable organic compounds containing both C and N preferentially, and decompose the C and N at the same time [81, 82]. Since the concentration of N and P in litter is usually lower than the requirements of microbes related to litter decomposition, microbes could reduce C utilization to adapt the decomposition environments with low-nutrient [36]. This might be the reason why the value of C release/N release ratio was lower than 1:1. On the other hand, microbes could also absorb inorganic N and P from the decomposing environment to maintain its stoichiometric balance and continue to decompose litter [34–36]. In our study, the concentrations of N and P in litter were the controlling factors of the coupling relationship of C and N release, which might be attribute to the restriction of N and P on litter decomposition.

The study revealed the influence of water level change and nutrient enrichment on the decomposition process of litter, and the role of litter properties and stoichiometry in controlling the nutrients dynamics during litter decomposition. Laboratory studies may not extrapolate well to natural systems, so, more experiments are needed to confirm the controlling mechanism of stoichiometry of litter and microbes on litter under different nutrient enrichment and water level treatments [26, 83]. The new index of real-time decomposition rate proposed in this study could be used to reveal the effects of other external factors or internal factors on litter decomposition and the relationship between litter nutrients and carbon sequestration in wetland.

## Conclusions

Our research found that water level change and nutrient enrichment significantly affected the litter decomposition and the dynamics of nutrients. The increase of water level reduced the decomposition rate of litter in the nutrient control treatment, while water level change made no significant difference in the nutrient enrichment treatment, indicating that the effect of water level change on litter decomposition could be affected by the nutrient enrichment. By comparing the real-time decomposition rates, our result demonstrated that the nutrient enrichment could accelerate litter decomposition and prolong the decomposition process under global climate change. Our results also showed that the decomposition of invasive plant litter and the coupling relationship of C and N release were limited by P. Therefore, controlling the P enrichment in water bodies is of great significance for the management of invasive plant and carbon sequestration of wetlands under the background of global climate change. Furthermore, these results provide a basis for evaluating the ecological functions of nutrient cycle and carbon sequestration in wetlands under global climate change. Additionally, the new index of real-time decomposition rate suggested in this study could be a supplementary of decomposition rate to reflect the influence of environmental factors on the entire litter decomposition process and to demonstrate the completeness of litter decomposition. Quantifying the new index of real-time decomposition rate could help to understand the decomposition process of invasive plant litter in the wetland ecosystems in chemical aspect, and also could provide a basis for quantifying and estimating the impacts of invasive plant litter decomposition on carbon cycle in wetlands.

## Supporting information

S1 Text The new index of real-time decomposition rate and the formula derivation. (DOCX)

S1 Table Details of the synthetic wastewater. (DOCX)

S2 Table Results of stepwise-regression analyses of litter nutrients release with the nutrient concentration and stoichiometric ratios of litter during the litter decomposition process. CC: C concentration, PC: P concentration, C/N: the ratio of C concentration to N concentration, C/P: the ratio of C concentration to P concentration.
(DOCX)

S3 Table Spearman correlations of real-time decomposition rate (K_i_) at the decomposition time i with the litter nutrient concentrations and stoichiometric ratios at time i-1 (C, N, P, C/N, N/P, and C/P ratios) tested in this experiment (n = 210). CC: C concentration, NC: N concentration, PC: P concentration, C/N: the ratio of C concentration to N concentration, N/P: the ratio of C concentration to N concentration, C/P: the ratio of C concentration to P concentration. **: *p* < 0.01, no superscript means *p* > 0.05.
(DOCX)

S1 Fig The relationship between N release and C release in 5cm (a), 15cm (b), and 25cm (c) water levels in the control treatment (Y1) and the nutrient enrichment treatment (Y2) during the experimental period. The blue and red areas are confidence intervals (95%).
(DOCX)
