# Supplementary material for: The decomposition process and nutrient release of invasive plant litter regulated by nutrient enrichment and water level change
Source: PLoS One. 2021 May 3;16(5):e0250880. doi: 10.1371/journal.pone.0250880 (PMC8092768; doi:10.1371/journal.pone.0250880)
Supplement: S1 Text — (DOCX) [file pone.0250880.s001.docx]

### S1 Text. The new index of real-time decomposition rate and the formula derivation.

The change rate of plant litter mass (M) for a discrete interval of time Δt can be expressed as:

$\frac{\Delta M}{\Delta t}=Income of interval-loss of interval$ (3)

The income of interval in the litterbag experiments is zero [51]. When ΔM and Δt approach 0, the limit value is the instantaneous change rate of plant litter mass:

$\frac{dM}{dt}=-KM$ (4)

where KM represents the loss of litter mass in the interval time Δt, K is considered as the instantaneous fractional loss rate.

And the formula (4) can be changed into:

$\frac{dM}{M}=-Kdt$ (5)

We integrated formula (5) on a semi-log graph of the remaining mass from an initial litter mass. To calculate the real-time decomposition rate (K_i_) at time i, we regarded time i-1 as the beginning of decomposition, and then

${lnM}_{i}=-K_{i}+lnM_{i-1}$ (6)

And the real-time decomposition rate (K_i_) was also fitted to the negative exponential decomposition model:

$\frac{M_{i}}{M_{i-1}}=e^{-K_{i}}$ (7)

where M_i_ represents the dry weight of litter remaining after i time of decomposition, M_i-1_ means the dry weight of litter remaining after i-1 time of decomposition, i and i-1 are the time points (day), K_i_ is regarded as the decomposition rate at time i.
