# Supplementary material for: The decomposition process and nutrient release of invasive plant litter regulated by nutrient enrichment and water level change
Source: PLoS One. 2021 May 3;16(5):e0250880. doi: 10.1371/journal.pone.0250880 (PMC8092768; doi:10.1371/journal.pone.0250880)
Supplement: S1 Table — (DOCX) [file pone.0250880.s002.docx]

**S1 Table. Details of the synthetic wastewater**

| Nutrients condition (mg/L) | COD | NH_4_^+^ –N | TN | TP |  |  |  |
| --- | --- | --- | --- | --- | --- | --- | --- |
|  | 60 | 8 | 20 | 1 |  |  |  |
| Ingredient concentration(mg/L) | Sucrose | (NH_4_)_2_SO_4_ | KNO_3_ | KH_2_PO_4_ | MgSO_4_ | CaCl_2_ | FeSO_4_ 7H_2_O |
|  | 51.345 | 37.6 | 76.57 | 7 | 10 | 10 | 18.289 |
