# Supplementary material for: The decomposition process and nutrient release of invasive plant litter regulated by nutrient enrichment and water level change
Source: PLoS One. 2021 May 3;16(5):e0250880. doi: 10.1371/journal.pone.0250880 (PMC8092768; doi:10.1371/journal.pone.0250880)
Supplement: S2 Table — CC: C concentration, PC: P concentration, C/N: the ratio of C concentration to N concentration, C/P: the ratio of C concentration to P concentration. (DOCX) [file pone.0250880.s003.docx]

**S2 Table. Results of stepwise-regression analyses of litter nutrients release with the nutrient concentration and stoichiometric ratios of litter during the litter decomposition process.** CC: C concentration, PC: P concentration, C/N: the ratio of C concentration to N concentration, C/P: the ratio of C concentration to P concentration.

|  | Regressions | R^2^ | F | *p* |
| --- | --- | --- | --- | --- |
| 1 | **C release=4.73CC-266.163PC+0.47C/N-0.035C/P-116.068** | 0.546 | 72.795 | <0.001 |
| 2 | **N release=4.65CC-276.214PC+1.568C/N-0.038C/P-134.966** | 0.694 | 136.305 | <0.001 |
| 3 | **P release=4.63CC-549.768PC +0.441C/N-0.046C/P -53.244** | 0.823 | 278.845 | <0.001 |
