# Supplementary material for: The decomposition process and nutrient release of invasive plant litter regulated by nutrient enrichment and water level change
Source: PLoS One. 2021 May 3;16(5):e0250880. doi: 10.1371/journal.pone.0250880 (PMC8092768; doi:10.1371/journal.pone.0250880)
Supplement: S3 Table — CC: C concentration, NC: N concentration, PC: P concentration, C/N: the ratio of C concentration to N concentration, N/P: the ratio of C concentration to N concentration, C/P: the ratio of C concentration to P concentration. **: p < 0.01, no superscript means p > 0.05. (DOCX) [file pone.0250880.s004.docx]

**S3 Table. Spearman correlations of real-time decomposition rate (K_i_) at the decomposition time i with the litter nutrient concentrations and stoichiometric ratios at time i-1 (CC, NC, PC, C/N, N/P, and C/P ratios) tested in this experiment (n = 210).** CC: C concentration, NC: N concentration, PC: P concentration, C/N: the ratio of C concentration to N concentration, N/P: the ratio of C concentration to N concentration, C/P: the ratio of C concentration to P concentration. **: *p* < 0.01, no superscript means *p* > 0.05.

|  | **K_i_** | **CC** | **NC** | **PC** | **C/N** | **N/P** | **C/P** |
| --- | --- | --- | --- | --- | --- | --- | --- |
| **CC** | -0.468** | 1 |  |  |  |  |  |
| **NC** | 0.103 | -0.184** | 1 |  |  |  |  |
| **PC** | 0.273** | -0.585** | 0.207** | 1 |  |  |  |
| **C/N** | -0.195** | 0.356** | -0.978** | -0.309** | 1 |  |  |
| **N/P** | -0.316** | 0.557** | 0.127 | -0.922** | -0.008 | 1 |  |
| **C/P** | -0.291** | .629** | -0.208** | -0.997** | 0.318** | 0.922** | 1 |
