# Supplementary material for: The decomposition process and nutrient release of invasive plant litter regulated by nutrient enrichment and water level change
Source: PLoS One. 2021 May 3;16(5):e0250880. doi: 10.1371/journal.pone.0250880 (PMC8092768; doi:10.1371/journal.pone.0250880)
Supplement: S1 Fig — The blue and red areas are confidence intervals (95%). (DOCX) [file pone.0250880.s005.docx]

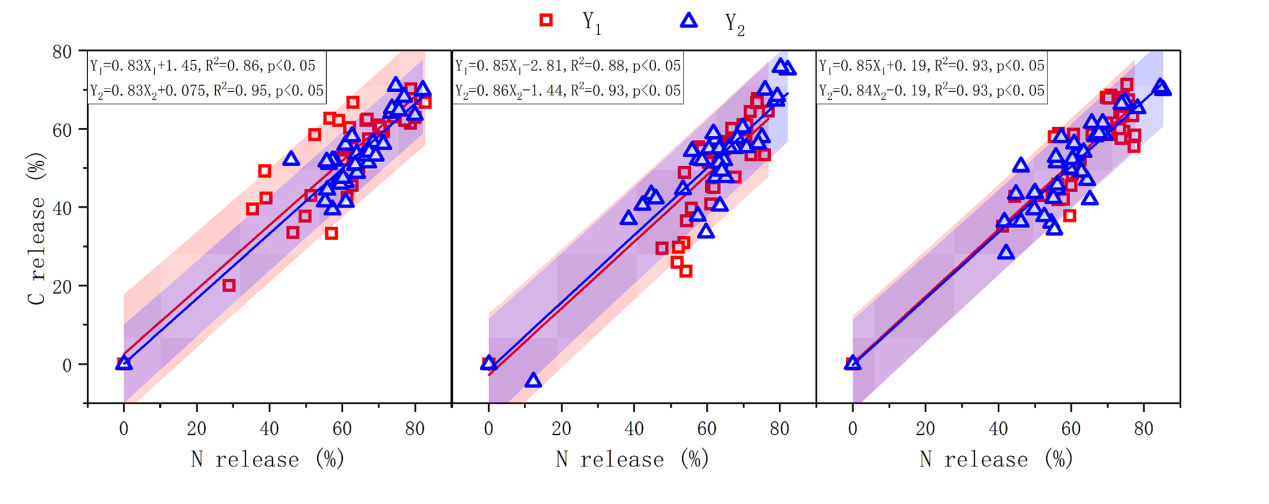


**S1 Fig. The relationship between N release and C release in 5cm (a), 15cm (b), and 25cm (c) water levels in the control treatment (Y1) and the nutrient enrichment treatment (Y2) during the experimental period.** The blue and red areas are confidence intervals (95%).
